# Correction: A mixed-methods multi-site case study of a person-centred intervention for constant observation in hospitals with people living with dementia

**DOI:** 10.1371/journal.pone.0349222

**Published:** 2026-05-11

**Authors:** Melanie Handley, Danai Theodosopoulou, Nicky Taylor, Rebecca Hadley, Claire Surr, Claire Goodman, Rosemary Phillips, Rowan H. Harwood

The following information is missing from the Funding statement: This study is supported by the National Institute for Health and Care Research (NIHR) Applied Research Collaboration East of England (NIHR ARC EoE) at Cambridgeshire and Peterborough NHS Foundation Trust. The views expressed are those of the author(s) and not necessarily those of the NIHR or the Department of Health and Social Care.

## References

[1] Handley M, Theodosopoulou D, Taylor N, Hadley R, Surr C, Goodman C, et al. A mixed-methods multi-site case study of a person-centred intervention for constant observation in hospitals with people living with dementia. PLoS One. 2025;20(10):e0321166. doi: 10.1371/journal.pone.0321166 41066362 PMC12510497

