# Supplementary material for: A mixed-methods multi-site case study of a person-centred intervention for constant observation in hospitals with people living with dementia
Source: PLoS One. 2025 Oct 9;20(10):e0321166. doi: 10.1371/journal.pone.0321166 (PMC12510497; doi:10.1371/journal.pone.0321166)
Supplement: S1 Table — (DOCX) [file pone.0321166.s001.docx]

Supplementary file 1, Table 1: Process for co-design meetings (CDM)

| **Stage** | **Development process** |
| --- | --- |
| CDM1 | Introduction of project, discussion and completion of paperwork including participant consent forms. |
| CDM2 | Skills audit of contributors to establish what people feel they can offer the process, rather than making assumptions or prescribing specific aspects of involvement. Goal setting by each participant to establish their motivation and to manage expectations. Goals can be revisited during process and at end to measure success. |
| Before CDM3 | Findings from phase 1 used to identify potential intervention components to address common priorities and organisational challenges to support PCC during constant observation across the sites. |
| CDM3 | Outline of potential priorities presented. Researchers facilitate group discussions to identify benefits and challenges in their local environment with aim of identifying priorities and agree way forward. |
| Between CDM 3 and CDM4 | Research team discuss findings from CDM3 and collate responses to identify priorities. |
| CDM4 | Cross-site meeting to present combined priorities across the three sites. Discussion and identification of the main focus of the proposed intervention. |
| Between CDM4 and CDM5 | Research team collate and agree the broad parameters for the intervention based on CDM4 feedback. Plan for implementation and testing feasibility and acceptability of the intervention is finalised. |
| CDM5, CDM6 & CDM7 | Site-specific co-design meetings with detailed progress of intervention design. Research team progress idea development between meetings. |
| Between CDM7 and CDM8 | Research team collate findings from three sites to produce one coherent vision for the intervention. |
| CDM8 | Cross-site meeting to present final version of intervention and address any final contributor concerns. Process for testing feasibility and acceptability is agreed. |
| Between CDM8 and CDM9 | Monthly newsletters or other accessible format updates to the co-design team during implementation phase. |
| CDM9 (Consensus event) | Final post-implementation cross-site consensus event to agree feasibility and acceptability of use of the intervention in practice. Acknowledgment and celebration of contributor roles. |
| CDM10 | Site specific debrief and project conclusion, revisit individual goals, establish interest in future dissemination activities |

CDM = Co-design meeting
