# Supplementary material for: A mixed-methods multi-site case study of a person-centred intervention for constant observation in hospitals with people living with dementia
Source: PLoS One. 2025 Oct 9;20(10):e0321166. doi: 10.1371/journal.pone.0321166 (PMC12510497; doi:10.1371/journal.pone.0321166)
Supplement: S2 TiDier Framework — (DOCX) [file pone.0321166.s002.docx]

Supplementary file 2: CONNECT Enhanced Care intervention reported according to the TiDier framework

| Brief Name and components  Provide the name or a phrase that describes the intervention. | CONNECT Enhanced care intervention   1. Making a Difference document 2. Patient Comfort booklet 3. Family and Friends leaflet 4. Peer Reflection tool |
| --- | --- |
| WHY  Describe any rationale, theory, or goal of the elements essential to the intervention. | Person-centred care with people living with dementia supported through constant observation practices during their admission to hospital can be difficult to achieve due to personal, interpersonal, service and organisational factors. The CONNECT Enhanced care intervention utilises focused staff time with patients (allocation to constant observation) to encourage care that can anticipate and address a person’s unmet needs, reducing distress and risk of an adverse event.  The intervention was designed to work within the existing practices, processes and structures of the ward and aid staff to use of person-centred approaches with people living with dementia who are identified as needing additional support through the use of constant observation (1:1 or cohorting practices). |
| WHAT  Materials: Describe any physical or informational materials used in the intervention, including those provided to participants or used in intervention delivery or in training of intervention providers. Provide information on where the materials can be accessed (e.g. online appendix, URL). | Each component was developed to address key aspects for a ward approach to person-centred constant observation with people living with dementia:   1. The Making a Difference document provides a framework for the considerations and possible actions during each period of Enhanced Care and to encourage sharing of information. 2. The Patient Comfort Booklet to record useful information about the person that can be drawn on during Enhanced Care and encourage communication across care partners. 3. The Family and Friends information leaflet to be handed to visitors by staff with the aim of raising awareness of Enhanced Care with family and friends of people with dementia assigned Enhanced Care, encourage sharing of information/strategies and manage expectations for care. 4. The Peer Reflection Tool to be used in brief development sessions with staff providing direct constant observation care. To encourage sharing of helpful strategies, positive experiences and challenging situations during Enhanced Care to consider ways to improve practice and learn from colleagues.   Training in the intervention. Staff on the study wards received training for how to use all components of the intervention. Printed guides for staff were made available in each of the wards.  Two implementation champions were identified on each ward and trained and supported by researchers to promote the use of the intervention with staff and to |
| WHAT  Procedures: Describe each of the procedures, activities, and/or processes used in the intervention, including any enabling or support activities. | 1. Making a Difference document: used as part of handovers to communicate a person’s needs, support goal setting and reflection. At the start of a period of Enhance Care, staff are encouraged to use the document to understand the purpose of Enhanced Care, consider how a person’s experience of care can be enhanced during that session, and, if a version of the document has already been completed for the person, to look for tips from colleagues about what has or has not worked. The document can also be used during Enhanced Care to record thoughts about the person’s care, for example what went well and tips for colleagues, a completed example can be found in this pack. This document can be left in a convenient place by the person’s bedside for colleagues to refer to if a handover has not been possible. 2. Patient Comfort Booklet: for staff to collect and share knowledge about the person with help from families/supporters, the person and other staff. Information can be added to the booklet as it is discovered, for example during conversations with the person, from colleagues who have worked with the person, from visitors or telephone conversations with people who support the person. Tips for information to include in each section are included on the back page of the booklet. 3. Family and Friends Information Leaflet: To be handed to visitors of people with dementia receiving Enhanced Care during their visits to the ward. Offering visitors the leaflet may be a new way of working with families and friends and may raise some concerns. Suggestions for how to do this were included as part of training. 4. Peer Reflection Tool: used in a number of ways depending on what works for the ward. One suggestion is to hold brief, small group sessions lead by someone knowledgeable in dementia care to work through an example(s). The questions on the poster can be used to guide the discussion. The discussion can focus on one question, a few or all depending on time and what the group would like to discuss. Smaller versions can also be used. |
| WHO PROVIDED  For each category of intervention provider (e.g. psychologist, nursing assistant), describe their expertise, background and any specific training given. | Staff providing direct care through constant observation activities: predominantly Clinical Support Workers, student nurses, bank and agency staff. Work closely with one person or a small group of people to support their safety and enhance their care. This is an opportunity to get to know the person. Trained in the use of all the materials.  Implementation champions: identified by ward manager to promote use of the intervention with colleagues. Training in all the materials and training and support in their role as implementation champion.  Ward managers: responsible for the work on the ward, identified how, when and by who the intervention would be used in the ward. Discussion with researchers about all the materials and staff who would be responsible for their use. |
| HOW  Describe the modes of delivery (e.g. face-to-face or by some other mechanism, such as internet or telephone) of the intervention and whether it was provided individually or in a group. | Researchers provided in-person and online training and drop in sessions with staff to explain the use of the materials.  Implementation Champions and Ward Managers promoted the use of the intervention at times when researchers were not on site.  Researchers provided additional, remote support for Implementation Champions between their visits to the ward.  Intervention materials were printed by the research team and provided to all the wards as pre-prepared packs.  Brief training videos introducing the intervention were created and shared via ward team messaging channels, such as WhatsApp. |
| WHERE  Describe the type(s) of location(s) where the intervention occurred, including any necessary infrastructure or relevant features. | The intervention was used in hospital wards. Locations for storing and use of the intervention were agreed locally. For example, wards decided whether Patient Comfort Booklets were attached to patient boards or kept in bedside folders with other care notes. |
| WHEN and HOW MUCH  Describe the number of times the intervention was delivered and over what period of time including the number of sessions, their schedule, and their duration, intensity or dose. | Taken from reports generated by Implementation Champions:  Over 12 week period, 34 reports from across six wards recorded the intervention was used 40 times. |
| TAILORING  If the intervention was planned to be personalised, titrated or adapted, then describe what, why, when, and how. | This stage of the study was to understand how the intervention might need to be adapted to local needs. This included ways that the intervention would be seen as legitimate documentation (e.g. spaces for labels and numbering systems), how the intervention was used in the wards (e.g. were documents displayed above a person’s bed for ease of reading or stored with other care notes), and whether there was adaptation of the original proposed used (e.g. 1. Prompt in handovers v as part of supervision of clinical support workers, 4. Brief peer support training opportunities v displayed in public ward areas as a reminder to staff) |
| MODIFICATIONS  If the intervention was modified during the course of the study, describe the changes (what, why, when, and how). | Not modified by research team. How staff used intervention was recorded, for example where intervention was kept and displayed, how staff used intervention as part of their daily work. |
| HOW WELL  Planned: If intervention adherence or fidelity was assessed, describe how and by whom, and if any strategies were used to maintain or improve fidelity, describe them. | Implementation Champions asked to keep a weekly record of use of intervention by other members of the team including number used, components used, by who.  Interviews, observations and researcher fieldnotes supplemented information from Implementation Champions. |
| HOW WELL  Actual: If intervention adherence or fidelity was assessed, describe the extent to which the intervention was delivered as planned. | 1. Evidence of use between clinical support workers providing 1:1 care regularly and senior nurses as a tool for brief, in-the-moment supervision to plan actions for care 2. Evidence of use by clinical support workers providing 1:1 care (completing documents with person living with dementia and family supporters, referring to contents and using to inform practice). Evidence of use by other ward staff (referring to contents and using to inform practice). 3. Some evidence of leaflet given to family supporters, but generally |
