# Supplementary material for: A mixed-methods multi-site case study of a person-centred intervention for constant observation in hospitals with people living with dementia
Source: PLoS One. 2025 Oct 9;20(10):e0321166. doi: 10.1371/journal.pone.0321166 (PMC12510497; doi:10.1371/journal.pone.0321166)
Supplement: S3 Table — (DOCX) [file pone.0321166.s003.docx]

Supplementary file 3

Table 1: Data collection schedule

| **Data collection** | **Participants** | **Baseline** | **Week 1/2** | **Week 6/7** | **Week 11/12** | **Mode of administration** |
| --- | --- | --- | --- | --- | --- | --- |
| NoMAD | Staff |  |  | X | X | Self or in conversation with researcher |
| Participant observation | Staff and people living with dementia and visitors to the ward | X | X | X | X | Researcher |
| Interviews | Staff |  |  | X | X | Researcher |
| Interviews | People living with dementia and family members |  | X | X | X | Researcher |
| CMAI-O* | People living with dementia | X | X | X | X | Researcher |
| SCIDS** | Staff | X |  |  | X | Self |
| QUIS*** | Staff and people living with dementia |  | X | X | X | Researcher |
| Field notes | Researcher reflections | Throughout | | | | Researcher |

*Cohen-Mansfield Agitation Inventory Observational (CMAI-O) (Griffiths et al., 2020) assesses agitation of participants by observations recorded by the researcher. The scale has been validated for use in care homes but to our knowledge has not been used in hospital studies.

**Sense of competence in dementia care staff (SCIDS) (Schepers et al., 2012) measures staffs’ perceived sense of competence in dementia. It is a self-administered, 17-item scale across four subscales (Professionalism, Building Relationships, Care Challenges, and Sustaining Personhood).

***The Quality of Interactions Schedule (QUIS) (Bridges et al., 2019) An observational tool for interactions between staff and patients. Originally developed for use in care homes, it has been used in hospital studies of patient care.

Bridges, J., Gould, L., Hope, J., Schoonhoven, L., & Griffiths, P. (2019). The Quality of Interactions Schedule (QuIS) and person-centred care: Concurrent validity in acute hospital settings. *International Journal of Nursing Studies Advances*, *1*, 100001.

Griffiths, A. W., Albertyn, C. P., Burnley, N. L., Creese, B., Walwyn, R., Holloway, I., Safarikova, J., & Surr, C. A. (2020). Validation of the Cohen-Mansfield agitation inventory observational (CMAI-O) tool. *International psychogeriatrics*, *32*(1), 75-85.

Schepers, A. K., Orrell, M., Shanahan, N., & Spector, A. (2012). Sense of competence in dementia care staff (SCIDS) scale: development, reliability, and validity. *International psychogeriatrics*, *24*(7), 1153-1162.
