# Supplementary material for: A mixed-methods multi-site case study of a person-centred intervention for constant observation in hospitals with people living with dementia
Source: PLoS One. 2025 Oct 9;20(10):e0321166. doi: 10.1371/journal.pone.0321166 (PMC12510497; doi:10.1371/journal.pone.0321166)
Supplement: S4 Tool — (DOCX) [file pone.0321166.s004.docx]

**Supplementary file 4: CONNECT Study Intervention Audit Tool**

Ward: Week:

To be completed by Implementation Champion [initials]

| 1. **Number of people with dementia assigned to constant observation this week (please provide numbers where possible** |  |
| --- | --- |
| 1. **Type of constant observation (numbers where possible)** | |
| 1:1 |  |
| Falls bay or Cohorted bay |  |
| Other |  |

| Making a Difference document | |
| --- | --- |
| 1. **Number used in this week** |  |
| 1. **Referred to in handover**   Yes  No  Don’t know | |
| 1. **Frequency of use in handover (numbers where possible)** | |
| More than 5 times a day |  |
| Between 3 and 5 times a day |  |
| Twice a day |  |
| Once a day |  |
| Never |  |
| Other | Once/twice in a week |
| 1. **Who typically was involved in handover (numbers where possible)?** | |
| Clinical Support Worker (ward staff) |  |
| Clinical Support Worker (enhancement team) |  |
| Student nurse |  |
| Bank/agency staff |  |
| Nurse |  |
| Ward manager |  |
| Other |  |

|  |  | |
| --- | --- | --- |
| Patient Comfort Booklet | | |
| 1. **Number completed** |  | |
| 1. **Who completed by (indicate number if possible)?** | | |
| Clinical Support Worker (ward staff) |  | |
| Clinical Support Worker (enhancement team) |  | |
| Student nurse |  | |
| Bank/agency staff |  | |
| Nurse |  | |
| Other |  | |
| 1. **Who completed with** **(indicate number if possible)? (one of the booklets was completed by the staff with the patient)** | | |
| Patient |  | |
| Family or other supporter |  | |
| Other members of staff |  | |
| Lone completion (e.g. staff completed based on their observation only) |  | |
| 1. **Sections completed (indicate number if possible)** | | |
| Communication |  | |
| Activities |  | |
| Sensory needs |  | |
| Reassurance |  | |
| Something unique to me |  | |
| What helps with my reason for constant observation |  | |
| 1. **Referred to during Constant observation?**   Yes  No  Don’t know | | |
| 1. **Referred to during handovers**   Yes  No  Don’t know | | |
| 1. **Where is the booklet kept?** | |  |

| Family and Friends Leaflet |  |
| --- | --- |
| 1. **Number handed out** |  |
| 1. **Who handed out (indicate number if possible)? (CSW and nurse working together so report numbers that reflect this – shared responsibility).** | |
| Clinical Support worker (ward staff) |  |
| Clinical support worker (enhancement team) |  |
| Student nurse |  |
| Bank / agency staff |  |
| Nurse |  |
| Ward manager |  |
| Other |  |
| 1. **Who handed to (indicate number if possible)?** | |
| Spouse/partner |  |
| Other family member |  |
| Friend |  |
| Other support (e.g. professional advocate). Please specify. |  |
| 1. **Follow up conversations**   Yes  No  Don’t know | |

| Peer Reflection Sessions | |
| --- | --- |
| 1. **Number completed** |  |
| 1. **Day and time** |  |
| 1. **How long for (minutes)?** |  |
| 1. **How many staff involved per session?** |  |
