# Supplementary material for: A mixed-methods multi-site case study of a person-centred intervention for constant observation in hospitals with people living with dementia
Source: PLoS One. 2025 Oct 9;20(10):e0321166. doi: 10.1371/journal.pone.0321166 (PMC12510497; doi:10.1371/journal.pone.0321166)
Supplement: S5 Table — (DOCX) [file pone.0321166.s005.docx]

Supplementary file 5

Table 1: Characteristics of participating wards

|  | | Ward A1 | Ward A2 | Ward B1 | Ward B2 | Ward C1 | Ward C2 |
| --- | --- | --- | --- | --- | --- | --- | --- |
| Ward type | | Older people’s medicine | Medical frailty | Older people’s medicine | Orthopedic | Older people’s admissions ward | Older people’s medicine |
| Number of beds | | 30 | 26 | 23 | 27 | 30 | 24 |
| Intended nurse staffing day  (Staff:patient ratio) | | 4 RN  4 CSW  (1:3.8) | 4 RN  4 CSW  (1:3.3) | 4 RN  4 CSW  (1:2.9) | 5 RN  4 CSW  (1:3) | 4 RN  6 CSW  (1:3) | 3 RN  5 CSW  (1:3) |
| Intended nurse staffing night  (Staff:patient ratio) | | 3 RN  3 CSW  (1:5) | 3 RN  3 CSW  (1:4.3) | 3 RN  3 CSW  (1:3.8) | 3 RN  4 CSW  (1:3.9) | 4 RN  5 CSW  (1:3.3) | 3 RN  4 CSW  (1:3.4) |
| Vacancies | | 0 | 1 RN | 1 RN  1 CSW | 1 RN | RNs and CSWs but no numbers | 5 RN  6 CSW |
| Regularity of temporary staff | | Two/ three times a week | Every shift | Two/ three times a week | Every shift | Every shift | Every shift |
| Regularity of 1-1 CO for patients with dementia | | Two/ three times a week | Every shift | Every shift | Every shift | Every shift | Two/ three times a week |
| Regularity of cohorting CO for patients with dementia | | Every shift | Every shift | Every shift | Every shift | Every shift | Every shift |
| Number of patients with dementia assigned CO in average week | | 3 (1-1)  3 (cohort) | 4 (1-1)  3 (cohort) | 3 (1-1)  3 (cohort) | Not reported (1-1)  5 or more (cohort) | 3 (1-1)  3 (cohort) | 1 (1-1)  3 (cohort) |
| Regularity of risks for people with dementia allocated CO | Falls | Often | Often | Yes but frequency not rated | Yes but frequency not rated | Very often | Very often |
|  | Trying to leave ward | Very often | Often | Yes but frequency not rated | Yes but frequency not rated | Sometimes | Sometimes |
|  | Pulling at IV | Often | Often | Yes but frequency not rated | Yes but frequency not rated | Often | Sometimes |
|  | Violence | Often | Often | Yes but frequency not rated | Yes but frequency not rated | Sometimes | Sometimes |
|  | Other | - | Anxiety/ depression - sometimes | Exploring other patients’ bed area and belongings | - | Self harm (sometimes) | Suicidal ideation – rarely |
| Previous involvement in research | | Yes | Yes | Yes | No | Yes | Yes |
