# Supplementary material for: A mixed-methods multi-site case study of a person-centred intervention for constant observation in hospitals with people living with dementia
Source: PLoS One. 2025 Oct 9;20(10):e0321166. doi: 10.1371/journal.pone.0321166 (PMC12510497; doi:10.1371/journal.pone.0321166)
Supplement: S6 Table — (DOCX) [file pone.0321166.s006.docx]

Supplementary file 6

A total of 39 staff completed NoMAD surveys. 29 completed week 6/7 surveys, 30 completed week 11/12 surveys with 20 staff completing both and 19 staff completing only one time point.

Responses for NPT construct questions were converted to numbers to calculate mean and median scores (strongly agree = 5, agree = 4, neither agree nor disagree = 3, disagree = 2, strongly disagree = 1)

Table 1: NoMAD Survey findings at timepoints 2 and 3

|  | W 6/7 (T2)  (n=29) | W 11/12 (T3)  (n=30) |
| --- | --- | --- |
| When you use CONNECT-EC how familiar does it feel? (1 low – 10 high) | Mean 6.2  Median 7  (range 1 – 10) | Mean 6.8  Median 7  (range 2 – 3) |
| Do you feel CONNECT-EC is currently a normal part of your work? (1 – 10) | Mean 6.2  Median 6  (range 1 – 10) | Mean 6.2  Median 6  (range 2 – 10) |
| Do you feel CONNECT-EC will become a normal part of your work? (1-10) | Mean 8.1  Median 8  (range 2 – 10) | Mean 7.8  Median 8  (range 1 – 10) |
| Coherence | | |
| I can see how CONNECT-EC differs from usual ways of working | *SA - 1  A - 22  N - 4  D - 0  SD - 0  NR – 1  (n=27) | SA - 6  A - 19  N - 4  D - 0  SD - 0  NR – 1  (n=29) |
| Staff in this organisation have a shared understanding of the purpose of CONNECT-EC | SA - 2  A - 17  N - 7  D - 0  SD - 0  NR – 2  (n=26) | SA - 8  A – 15  N - 5  D – 2  SD - 0  NR - 0 |
| I understand how CONNECT-EC affects the nature of my own work | SA - 2  A - 22  N – 2  D - 1  SD - 0  NR – 1  (n-27) | SA - 9  A - 19  N - 2  D - 0  SD - 0  NR – 0 |
| I can see the potential value of CONNECT-EC for my work | SA - 6  A - 18  N - 4  D - 0  SD - 0  NR – 0  (n=28) | SA - 16  A - 11  N - 3  D - 0  SD - 0  NR – 0 |
| Cognitive Participation | | |
| There are key people who drive CONNECT-EC forward and get others involved | SA - 4  A - 21  N - 3  D - 0  SD - 0  NR – 0  (n=28) | SA - 6  A - 20  N - 3  D – 1  SD - 0  NR – 0 |
| I believe that participating in CONNECT-EC is a legitimate part of my role | SA – 3  A – 22  N – 2  D - 0  SD - 0  NR – 1  (n=27) | SA - 9  A – 18  N - 1  D - 1  SD - 0  NR – 1  (n=29) |
| I’m open to working with colleagues in new ways to use CONNECT-EC | SA - 5  A - 21  N – 2  D - 0  SD - 0  NR – 0  (n=28) | SA - 11  A - 18  N - 1  D – 0  SD - 0  NR – 0 |
| I will continue to support CONNECT-EC | SA - 7  A - 20  N - 1  D - 0  SD - 0  NR – 0  (n=28) | SA - 16  A - 13  N – 0  D – 0  SD - 0  NR – 1  (n=29) |
| Collective action | | |
| I can easily integrate CONNECT-EC into my existing work | SA - 3  A – 23  N - 1  D - 0  SD - 0  NR – 1  (n=27) | SA - 7  A - 16  N – 6  D – 1  SD - 0  NR – 0 |
| CONNECT-EC disrupts working relationships | SA - 0  A – 3  N – 6  D – 18  SD – 1  NR – 0  (n=28) | SA - 2  A – 1  N – 2  D – 20  SD – 5  NR – 0 |
| I have confidence in other people’s ability to use CONNECT-EC | SA – 4  A – 13  N – 7  D – 2  SD – 0  NR – 1  (n=26) | SA – 4  A – 14  N – 11  D – 1  SD – 0  NR – 0 |
| Work is assigned to those with skills appropriate to CONNECT-EC | SA - 0  A – 13  N – 12  D – 2  SD - 0  NR - 1  (n=27) | SA - 4  A – 15  N – 8  D – 1  SD – 1  NR - 0  (n=29) |
| Sufficient training is provided to enable staff to implement CONNECT-EC | SA – 2  A – 20  N – 1  D – 5  SD - 0  NR – 0  (n=28) | SA - 6  A - 10  N - 9  D - 4  SD - 0  NR – 1  (n=29) |
| Sufficient resources are available to support CONNECT-EC | SA - 1  A – 21  N – 2  D – 4  SD - 0  NR – 0  (n=28) | SA - 4  A - 17  N - 6  D – 2  SD - 0  NR – 1  (n=29) |
| Management adequately supports CONNECT-EC | SA – 2  A - 22  N - 3  D - 1  SD - 0  NR – 0  (n=28) | SA - 8  A – 15  N - 6  D - 0  SD - 0  NR – 1  (n=29) |
| Reflexive Monitoring | | |
| The staff agree that CONNECT-EC is worthwhile | SA - 3  A – 19  N – 5  D – 1  SD - 0  NR – 1  (n=28) | SA – 6  A - 16  N - 8  D - 0  SD - 0  NR – 0 |
| I value the effects that CONNECT-EC has on my work | SA - 5  A - 20  N - 2  D - 0  SD - 0  NR – 1  (n=27) | SA - 7  A - 20  N – 2  D - 0  SD - 0  NR – 0  (n=29) |
| Feedback about CONNECT-EC can be used to improve it in the future | SA - 6  A - 21  N – 1  D – 0  SD - 0  NR – 0  (n=28) | SA - 10  A – 20  N - 0  D - 0  SD - 0  NR – 0 |
| I can modify how I work with CONNECT-EC | SA - 6  A - 21  N - 0  D - 0  SD - 0  NR – 1  (n=27) | SA - 10  A - 16  N - 4  D - 0  SD - 0  NR – 0 |

*SA – Strongly Agree, A – Agree, N – Neither Agree or Disagree, D – Disagree, SD – Strongly Disagree, NR – Not Relevant
