# Supplementary material for: A mixed-methods multi-site case study of a person-centred intervention for constant observation in hospitals with people living with dementia
Source: PLoS One. 2025 Oct 9;20(10):e0321166. doi: 10.1371/journal.pone.0321166 (PMC12510497; doi:10.1371/journal.pone.0321166)
Supplement: S7 Table — (DOCX) [file pone.0321166.s007.docx]

Table 1: Good reporting of a mixed methods study checklist

| Item | Section and page number |
| --- | --- |
| Describe the justification for using a mixed methods approach to the research question | Methods, page 6 |
| Describe the design in terms of the purpose, priority and sequence of methods | Methods, page 6 |
| Describe each method in terms of sampling, data collection and analysis | Methods, page 10 - 16 |
| Describe where integration has occurred, how it has occurred and who has participated in it | Methods, page 16 |
| Describe any limitation of one method associated with the present of the other method | Limitations, p36 |
| Describe any insights gained from mixing or integrating methods | Limitations, p36 |

Methods, Page 6–7 2 Methods, Page 6–7 3 Methods, Page 7–11 4 Discussion, Page 18–21 5 Discussion, Page 18–21 6
